# In-Hospital Outcomes of Patients With Non-Alcoholic Fatty Liver Disease Who Underwent Percutaneous Coronary Intervention: A Nationwide Inpatient Sample Analysis

**DOI:** 10.7759/cureus.17338

**Published:** 2021-08-20

**Authors:** Hasan Ali, Maryam Kazmi, Catherine Choi, Reza Hashemipour, Inderjit Singh, Nikolaos T Pyrsopoulos

**Affiliations:** 1 Internal Medicine, Rutgers University New Jersey Medical School, Newark, USA; 2 Gastroenterology and Hepatology, Rutgers University New Jersey Medical School, Newark, USA; 3 Gastroenterology and Hepatalogy, Rutgers University New Jersey Medical School, Newark, USA; 4 Cardiology, Rutgers University New Jersey Medical School, Newark, USA

**Keywords:** primary percutaneous coronary intervention (pci), nonalcoholic fatty liver disease (nafld), coronary artery disease, atherosclerosis, non-alcoholic steatohepatitis (nash), metabolic syndrome

## Abstract

Background

Non-alcoholic fatty liver disease (NAFLD) is prevalent in almost 25% of the Western population and is predicted to become one of the leading causes of end-stage liver disease. There is increasing evidence that NAFLD is a risk factor for cardiovascular disease, specifically for coronary artery disease, via disruption of the metabolism of glucose and lipids in the body, leading to a state of systemic inflammation that promotes atherosclerosis. This study aims to explore outcomes in patients who underwent percutaneous coronary intervention (PCI) with or without placement of drug-eluting stents (DES) to determine whether the concurrent diagnosis of NAFLD led to worse in-hospital outcomes.

Methods

We used the National Inpatient Sample, Healthcare Cost and Utilization Project (HCUP), Agency for Healthcare Research and Quality 2016 to conduct a cross-sectional study that included all adult patients who underwent PCI with or without placement of DES during hospital admission. Patients with NAFLD were identified and compared to patients without NAFLD. Patients were selected by using ICD-10-CM and ICD-10-PCS codes. Outcomes included mortality, length of stay and total hospital charges, and major adverse cardiac events (MACE). Data on patient demographics, inpatient statistics, and comorbidities were obtained and analyzed using cross-tabulation, Pearson χ^2^ test, and independent samples *t*-test. Data were adjusted for confounders using logistic and linear regression.

Results

Among 429,855 patients who underwent PCI with or without placement of DES, 2,560 patients (0.6%) had a diagnosis of NAFLD. There was no significant difference with regard to mortality and MACE. The NAFLD group had a higher proportion of females, a longer average length of hospital stay, and patients presented at a younger average age. Regarding comorbidities, more patients in the NAFLD group had diabetes mellitus type II, obesity, obstructive sleep apnea (OSA), chronic kidney disease (CKD), and peripheral vascular disease (PVD).

Conclusion

NAFLD is emerging as a risk factor for cardiovascular disease. Increasing evidence suggests that the disease contributes to systemic atherosclerosis and thus coronary artery disease. We found that among patients who underwent PCI in 2016, those with NAFLD had a longer length of stay, were admitted at a younger age, and had significantly more cardiovascular comorbidities than those without NAFLD. Increasing evidence has shown that advanced liver disease due to NAFLD will continue to place a significant burden on the healthcare system and is, therefore, an area that the medical community should continue to focus on, especially, regarding preventative and therapeutic efforts.

## Introduction

Non-alcoholic fatty liver disease (NAFLD) is prevalent in almost 25% of the Western population and is predicted to become one of the leading causes of end-stage liver disease in the years to come [1,2]. NAFLD is a broad diagnosis that solely indicates fatty infiltration of the liver; however, it can progress to non-alcoholic steatohepatitis (NASH) which indicates fatty infiltration causing hepatocyte injury and inflammation. NASH can eventually lead to liver cirrhosis and end-stage liver disease, necessitating the need for intensive medical care and eventually liver transplantation [2]. Thus, the fatty liver disease poses a serious threat as a significant burden to our healthcare system.

Cardiovascular disease is one of the leading causes of morbidity and mortality in the world. It is important to identify various subgroups of the population who are at higher risk of cardiovascular disease and its complications. There is increasing evidence that NAFLD is a risk factor for cardiovascular disease, specifically for coronary artery disease (CAD). The pathogenesis of NAFLD involves disruption of the metabolism of glucose and lipids in the body, leading to a state of systemic inflammation and an increased risk of developing atherosclerosis [1]. NAFLD is also associated with increased stiffness of arteries and a higher number of cardiovascular events when compared to the non-NAFLD population [2]. As growing evidence continues to support the association between NAFLD and CAD, further investigation is necessary to determine the relevant outcomes of these patients. A study by Keskin et al. explored the outcomes of 360 patients with ST-elevation myocardial infarction (STEMI) with or without NAFLD and found that the patients with NAFLD experienced higher in-hospital mortality risk when compared to those without NAFLD [3]. They also noted that increasing severity of NAFLD correlated with higher mortality risk in STEMI patients. Another study by Xia et al. explored the outcomes of elderly patients with NAFLD who suffered an acute myocardial infarction, and their findings supported the notion that patients with NAFLD tend to have worse cardiovascular outcomes and increased mortality when compared to those without NAFLD [4]. 

Our goal was to use the Nationwide Inpatient Sample (NIS) database of patients from 2016 to explore outcomes in patients who underwent percutaneous coronary intervention (PCI) to determine whether the concurrent diagnosis of NAFLD led to worse in-hospital outcomes. Given the pathogenesis of NAFLD and the mounting evidence for its role in the development of CAD, we hypothesized that in-hospital mortality, hospital length of stay, and cardiovascular complications would be higher in the group that had NAFLD. Also, when considering NAFLD as part of the presentation of metabolic syndrome, we would expect rates of obesity, diabetes mellitus, and other related comorbidities to be higher in this group.

## Materials and methods

Data source

The National Inpatient Sample (NIS) database was developed for the Healthcare Cost and Utilization Project (HCUP) and maintains data on 20% of all inpatient admissions at non-federal hospitals. The NIS is a product of the Agency for Healthcare Research and Quality and contains patient information which has been de-identified. The database is a nationally representative subset acquired through hospital discharge records and is the largest in-patient database currently available in the United States. The database includes about seven million inpatient hospital admissions and stratifies all-payer inpatient data by type of insurance and hospital region [5]. The database includes data pertaining to demographics, admission diagnoses, procedures/operations, cost, length of stay, and mortality. The 2016 database was queried for patients who were admitted to the hospital and underwent PCI with or without placement of a drug-eluting stent (DES), using the International Classification of Diseases-Tenth Edition Revision Clinical Modification (ICD-10-CM) and Procedure Coding System (ICD-10-PCS). While a proportion of the national population has been sampled, yearly sampling weights are applied which then provide national estimates. Years of data and a multitude of works have verified the value of this sampling tool [5].

Study design and inclusion criteria

This is a retrospective cross-sectional study and includes all patients greater than 18 years of age who underwent PCI with or without placement of DES during hospital admission in the year 2016. A total of 40 ICD-10-PCS codes were used to identify all patients who underwent PCI of one, two, three, or four or more coronary arteries or branches including bifurcations of arteries, with or without placement of up to four or more DES. Next, patients with a diagnosis of NAFLD were identified using the ICD-10-CM code “K76.0.” We divided the population into two groups: patients with a diagnosis of NAFLD, namely the NAFLD group, and the patients without a diagnosis of NAFLD, the non-NAFLD group. Patient-level and hospital-level weights provided in the dataset were used to generate population estimate samples. Primary outcomes for the study included mortality, length of stay, and hospital charges accrued. Secondary outcomes included incidence of one or more of five major adverse cardiac events (MACE), namely postprocedural cardiogenic shock, new-onset heart failure, new-onset atrial fibrillation, acute cerebrovascular accident, and cardiac arrest, which were identified using ICD-10-CM codes. Various patient demographics including age at the time of admission, race, sex, and elective versus non-elective procedure were obtained. Data on comorbidities, especially risk factors for developing cardiovascular disease, were obtained and compared between the two groups. ICD-10-CM codes were used to identify comorbidities included in the analysis, namely hypertension (HTN), dyslipidemia (HLD), type 2 diabetes with and without complications, chronic kidney disease (CKD), obesity, obstructive sleep apnea (OSA), pulmonary HTN, tobacco use, history of myocardial infarction, heart failure, peripheral vascular disease (PVD), and gastroesophageal reflux disease (GERD).

Statistical analysis

IBM SPSS Statistics, version 27 (IBM Corp., Armonk, NY), was used for all statistical analyses. Categorical and continuous variables were analyzed, and the results were used to identify significant differences between the NAFLD and non-NAFLD groups. Categorical variables were analyzed using descriptive frequencies, cross-tabulation, and Pearson χ^2^ test. Continuous variables were analyzed using independent samples t-test. Univariate and multivariate logistic regression models were used to analyze the association between predictor variables and outcomes of interest. The multivariate logistic regression models were adjusted for potential confounders, including demographic factors (i.e., age, sex, race) and comorbidities (pulmonary HTN, OSA, obesity, CKD, HLD, HTN, tobacco use, type 2 diabetes mellitus, heart failure, PVD, and prior history of myocardial infarction). Non-adjusted and adjusted odds ratios, confidence intervals, and p-values were reported where appropriate. Hypothesis testing was two-sided, and statistical significance was set at a p-value of ≤0.05.

## Results

Using the NIS database, 429,855 patients were found to have undergone PCI with or without placement of DES in the year 2016. Of these, 2,560 patients (0.6%) had a diagnosis of NAFLD (NAFLD group) and 427,295 patients did not have NAFLD (non-NAFLD group). Demographic data are recorded in Tables 1-2. In the NAFLD population, 35.7% were female, whereas 32.9% of the non-NAFLD population was female (OR 1.136, CI 1.048 to 1.232, p=0.002; aOR 1.255, CI 1.04 to 1.514, p=0.018). The race category of the patients was also identified, with both groups being majority Caucasian. The NAFLD group was more likely to have non-elective PCI procedures, however, when adjusted for age, sex, race, and multiple comorbidities, this difference was no longer statistically significant (OR 0.733, 95% CI 0.635 to 0.846, p<0.001; aOR 0.767, 95% CI 0.554 to 1.062, p=0.11).

**Table 1:** Demographics NAFLD: non-alcoholic fatty liver disease; N: sample size.

Variable	Percentage, NAFLD group (N=2560)	Percentage, Non-NAFLD group (N=427295)	Odds ratio	Confidence interval	p-Value	Adjusted odds ratio	Adjusted confidence interval	Adjusted p-Value
Sex - female	35.7	32.9	1.136	1.048-1.232	0.002	1.255	1.04-1.514	0.018
Elective procedure	8	10.6	<0.001	0.733	0.635-0.846	0.767	0.554-1.062	0.11
Non-elective procedure	92	89.4	<0.001	-	-	-	-	-

**Table 2:** Race demographics NAFLD: non-alcoholic fatty liver disease; N: sample size.

Race	NAFLD group		Non-NAFLD group		Total N
	N	Percentage	N	Percentage	
Caucasian	1745	70.5	308,475	75.6	310,220
Black	210	8.5	38,765	9.5	38,975
Hispanic	330	13.3	32,370	7.9	32,700
Asian	80	3.2	10,915	2.7	10,995
Native American	30	1.2	2305	0.6	2335
Other	80	3.2	15,000	3.7	15,080

Considering the primary outcome of mortality, there was no statistically significant difference between the NAFLD group and the non-NAFLD group (OR 0.781, 95% CI 0.581 to 1.049, p=0.10; aOR 1.11, 95% CI 0.570 to 2.164, p=0.757). The incidences of MACE were also measured using five parameters, which included postprocedural cardiogenic shock, new-onset heart failure, new-onset atrial fibrillation, acute cerebrovascular accident, and cardiac arrest. Incidences of 5-point MACE were not significantly different between the NAFLD and non-NAFLD group (OR 0.931, 95% CI 0.733 to 1.181, p=0.554; aOR 1.115, 95% CI 0.653 to 1.905, p=0.69). These findings are recorded in Table 3.

**Table 3:** Mortality and MACE compared between NAFLD and non-NAFLD groups NAFLD: non-alcoholic fatty liver disease; N: sample size; MACE: major adverse cardiac events.

Variable	Percentage, NAFLD group (N=2560)	Percentage, non-NAFLD group (N=427,295)	Odds ratio	Confidence interval	p-Value	Adjusted odds ratio	Adjusted confidence interval	Adjusted p-value
Mortality	1.8	2.2	0.1	0.781	0.581–1.049	1.11	0.570–2.164	0.757
5-point MACE	2.7	2.9	0.554	0.931	0.733–1.181	1.115	0.653–1.905	0.69

The NAFLD group demonstrated a longer average length of hospital stay versus the non-NAFLD group (mean 4.65±6.48 days versus 3.71±4.41 days, respectively), and the difference was statistically significant (p=0.001, 95% CI: 3.47 to 4.32; aOR 1.023, 95% CI: 1.007 to 1.04, p=0.005). There was also a statistically significant difference in the average age at the time of hospital admission, with the NAFLD group presenting at a mean age of 61.0±10.93 years and the non-NAFLD group presenting at 64.9± 12.3 years (p<0.001, 95% CI: −1.19 to −0.69; aOR 0.97, 95% CI: 0.963 to 0.978, p<0.001). The total charges incurred during hospitalization were also significantly higher for the NAFLD group, with a mean cost of $115,925±94,497 versus mean cost of $103,999±89,428 for the non-NAFLD group (p<0.001, CI: −15675.94 to −8175.26), however, once adjusted for confounders, there was no statistically significant difference (aOR 1.0, CI: −20334.96 to −3516.30, p=0.664). These findings are recorded in Table 4.

**Table 4:** Hospital admission data NAFLD: non-alcoholic fatty liver disease; LOS: length of stay; US: United States.

Variable	NAFLD group			Non-NALFD group			p-value	Mean difference	Confidence interval	Adjusted odds ratio	Adjusted confidence interval	Adjusted p-value
	Mean	Standard deviation	Standard error mean	Mean	Standard deviation	Standard error						
Age at admission	61	10.93	0.483	64.89	12.34	0.042	<0.001	3.893±0.485	2.94–4.85	0.97	0.963–0.978	<0.001
LOS (days)	4.65	6.48	0.287	3.71	4.41	0.015	0.001	−0.941±0.287	−1.51 to −0.377	1.023	1.007–1.04	0.005
Total Charges (US Dollars)	115,925	94,497	4268.95	103,999	89,428	308.01	0.006	−11925.63±4280.04	−20334.96 to −3516.30	1	1.0–1.0	0.664

With regards to comorbidities (Table 5), more patients in the NAFLD group had the following comorbidities when compared to the non-NAFLD group: diabetes mellitus type 2 (DMT2, 47.1% versus 36.7%, OR 1.531, 95% CI: 1.086 to 1.405, p<0.001; aOR 1.32, 95% CI: 1.099 to 1.587, p=0.003), HTN (64.5% versus 59.5%, OR 1.234, 95% CI: 1.138 to 1.339, p<0.001; aOR 1.307, 95% CI 1.042 to 1.639, p=0.02), obesity (23.6% versus 12.3%, OR 2.212, 95% CI: 2.019 to 2.425, p<0.001; aOR 1.738, 95% CI: 1.401 to 2.157, p<0.001), OSA (16.6% versus 8.7%, OR 2.099, 95% CI: 1.890 to 2.33, p<0.001; aOR 1.825, 95% CI: 1.427 to 2.333, p<0.001), CKD (15.2% versus 15%, OR 1.019, 95% CI: 0.915 to 1.136, p=0.729; aOR 1.422, 95% CI: 1.027 to 1.969, p=0.034), PVD (10.2% versus 8.4%, OR: 1.236, 95% CI 1.086 to 1.405, p=0.001; aOR 1.388, 95% CI: 1.031 to 1.868, p=0.031), and GERD (29.7% versus 18.5%, OR 1.864, 95% CI: 1.72 to 2.03, p<0.001; aOR 1.92, 95% CI: 1.578 to 2.337, p<0.001). Upon adjusting for confounders, there was no statistically significant difference between the NAFLD and non-NAFLD groups with regards to pulmonary HTN, HLD, history of prior myocardial infarction (MI), acute STEMI, acute non-ST-elevation myocardial infarction (NSTEMI), and congestive heart failure (CHF). There were less patients who smoked tobacco in the NAFLD group compared to the non-NAFLD group (1% versus 2.4%, OR 0.404, 95% CI: 0.273 to 0.6, p<0.001; aOR 0.308, 95% CI: 0.115 to 0.825, p=0.019).

**Table 5:** Comorbidities associated with NAFLD and non-NAFLD group NAFLD: non-alcoholic fatty liver disease; N: sample size; HTN: hypertension; GERD: gastroesophageal reflux disease; OSA: obstructive sleep apnea; CKD: chronic kidney disease; HLD: dyslipidemia; MI: myocardial infarction; NSTEMI: non-ST elevation myocardial infarction; STEMI: ST-elevation myocardial infarction; CHF: congestive heart failure; PVD: peripheral vascular disease; T2DM: diabetes mellitus type 2.

Comorbidities	NAFLD group		Non-NAFLD group		Odds ratio	Confidence interval	p-Value	Adjusted odds ratio	Adjusted confidence interval	Adjusted p-value
	N	Percentage	N	Percentage						
Pulmonary HTN	110	4.3	14,890	3.5	1.244	1.027–1.506	0.026	1.275	0.805–2.02	0.3
GERD	760	29.7	78910	18.5	1.864	1.71–2.030	<0.001	1.92	1.578–2.337	<0.001
OSA	425	16.6	37,015	8.7	2.099	1.890–2.33	<0.001	1.825	1.427–2.333	<0.001
Obesity	605	23.6	52,435	12.3	2.212	2.019–2.425	<0.001	1.738	1.401–2.157	<0.001
CKD stages 1–5	390	15.2	64,050	15	1.019	0.915–1.136	0.729	1.422	1.027–1.969	0.034
HLD	1670	65.2	271,855	63.3	1.073	0.989–1.164	0.091	0.981	0.811–1.186	0.841
Tobacco use	25	1	10.17	2.4	0.404	0.273–0.6	<0.001	0.308	0.115–0.825	0.019
HTN	1,650	64.5	254,225	59.5	1.234	1.138–1.339	<0.001	1.307	1.042–1.639	0.02
History of prior MI	455	17.8	72,160	16.9	1.064	0.961–1.178	0.233	1.023	0.81–1.291	0.85
NSTEMI (acute)	1145	44.7	175,900	41.2	1.156	1.07–1.25	<0.001	1.143	0.956–1.367	0.143
STEMI (acute)	60	2.3	11,780	2.8	0.847	0.655–1.094	0.203	0.843	0.462–1.537	0.577
CHF	575	22.5	95,310	22.3	1.009	0.919–1.107	0.851	1.074	0.864–1.336	0.519
PVD	260	10.2	35,815	8.4	1.236	1.086–1.405	0.001	1.388	1.031–1.868	0.031
T2DM	1,205	47.1	156,975	36.7	1.531	1.417–1.655	<0.001	1.32	1.099–1.587	0.003

## Discussion

Non-alcoholic fatty liver disease is emerging as a risk factor for cardiovascular disease. Increasing evidence supports the theory that NAFLD causes an increase in liver-secreted cytokines, procoagulant factors, and adhesion molecules, leading to a state of chronic inflammation that contributes to systemic atherosclerosis and thus coronary artery disease [1,6]. Previously, Keskin et al. reported that in patients with STEMI, the presence of NAFLD led to worse clinical outcomes. They also found that the higher the grade of NAFLD, the higher rate of mortality in STEMI patients [3]. We aimed to study outcomes of patients with NAFLD who underwent PCI to determine whether they had increased mortality or incidence of complications compared to patients without NAFLD. This study is the first large-scale retrospective cohort study to compare in-hospital PCI outcomes between these two groups. 

Our study included all patients in the 2016 NIS database who underwent PCI, with or without DES placement. The selected patients were then divided into groups based on whether or not they had NAFLD. Of note, from the 429,855 patients who underwent PCI in this population, only 2,560, or 0.6%, had a diagnosis of NAFLD. This likely represents a gross underestimation of the actual number of patients with NAFLD, as the disease is known to be underdiagnosed. In the Western population, the prevalence of NAFLD is estimated to be 20-30%, increases to 40-70% in patients with type 2 diabetes mellitus, and up to 90% of patients with morbid obesity [7]. There are multiple explanations for the discrepancy between estimates and recorded data, including lack of guidelines recommending routine screening for NAFLD, under-recognition of the diagnosis, or lack of confidence on the part of physicians in establishing the diagnosis based on the available data [7].

In this study, primary outcomes of interest included mortality, length of hospital stay, and total hospital charges. Our data demonstrated that NAFLD did not significantly affect in-hospital mortality in patients who underwent PCI, and there was no statistically significant difference in the total hospital charges incurred between the two groups when adjusted for confounding variables. However, patients with NAFLD who underwent PCI were hospitalized for a longer period of time than those without NAFLD. NAFLD patients may have had longer recovery times post-PCI due to the impact of comorbidities, including diabetes and CKD, or differences in nutritional status [8]. Future studies can be done to examine the variables that impact hospital length of stay in NAFLD patients. 

The NAFLD patients were also noted to be hospitalized at a significantly younger age than the non-NAFLD patients. This is a concerning finding as it may indicate that patients with NAFLD are more prone to developing early atherosclerosis of coronary arteries, putting them at risk of myocardial ischemia at a younger age [1,2,6,9,10]. Further research can be done to study NAFLD's impact on the age at onset of subclinical and clinical atherosclerosis. A correlation could change the clinical perception of NAFLD and promote early recognition and intervention to prevent the development and progression of atherosclerosis [11]. Although not statistically significant, patients with NAFLD were also more likely to be hospitalized for emergent or non-elective PCI procedures. This may indicate that patients with NAFLD are more likely to present with acute coronary syndromes requiring emergent intervention [3,6]. It may also reinforce the importance of outpatient screening for CAD in this population [11,12].

Notably, in our study, the NAFLD group had a greater burden of significant comorbidities, including obesity, diabetes mellitus, and OSA. Thus, it is consistent with the pattern seen in metabolic syndrome. When considering the pathophysiology of NAFLD, including insulin resistance, systemic inflammation, and predisposition in patients who are obese, our data are congruent with expected correlations with regards to such comorbidities [1,2,6,13]. Our study also revealed a higher burden of GERD, CKD, and PVD in the NAFLD population. Although these comorbidities confer a higher risk of atherosclerosis and CAD in patients with NAFLD, several studies show that after adjustment for other CAD risk factors, NAFLD remained an independent risk factor for CAD [2,6,14]. Other studies have shown a bidirectional relationship between NAFLD and risk factors for CAD, including diabetes and HTN [15]. 

One limitation of this study is that NAFLD is underdiagnosed in the selected population, which likely leads to an underestimation of the complications found in this group [2,12]. As mentioned earlier, NAFLD can be easily missed as it is an asymptomatic condition, is not always associated with elevations in liver function tests, and there are no formal screening guidelines. Thus, our results are not indicative of the actual disease burden in the population but instead capture a small subset of patients already diagnosed with NAFLD. Another limitation is that we did not stratify our NAFLD patients into groups based on severity, as was done in other studies [1,3,16]. Ultrasonography can be used to determine the degree of NAFLD based on echogenicity; however, this information is not available in the NIS database. It would be worthwhile to conduct additional studies to determine if the severity of fatty liver disease correlates with worse outcomes in patients who undergo PCI.

Since our study included all patients who underwent PCI with or without DES placement, additional investigations are necessary to elucidate whether there are significant differences between the patients who had stents placed and those who did not. Presumably, stent placement would indicate a more significant degree of coronary artery stenosis in most cases. Thus, stratifying patients based on the severity of coronary artery disease and then studying the effect of NAFLD on outcomes may reveal further relationships between these disease processes. Lu et al. demonstrated that end-stage liver disease patients who had successful PCI with stent placement had increased adverse in-hospital outcomes compared to those who were managed medically, but no difference in one-year mortality [17]. It would be of interest to perform a parallel study observing treatment-related outcomes in patients with NAFLD. 

Given the increasing worldwide incidence of liver disease due to NAFLD, it is imperative to understand the implications of this diagnosis. Early diagnosis and aggressive risk factor modification may help to prevent the long-term consequences of systemic inflammation, including atherosclerosis. Patients with NAFLD may also benefit from vigilant surveillance for coronary artery disease and more intensive therapeutic interventions [9]. A growing number of studies are investigating the use of pharmacologic therapy to minimize the effects of NAFLD, such as vitamin E, statins, and thiazolidinediones [18,19]. Longitudinal studies still need to be done to determine if these interventions will produce changes in outcomes.

## Conclusions

Overall, though data from this specific patient population did not indicate that NAFLD increases in-hospital mortality, there were still significant differences between the NAFLD and non-NAFLD groups. We found that among patients who underwent PCI in 2016, those with NAFLD had a longer length of stay, were admitted at a younger age, and had significantly more cardiovascular comorbidities than those without NAFLD. When considered in the context of extensive novel data identifying NAFLD as an independent risk factor for coronary artery disease, these differences may represent practice-changing findings. Increasing evidence has shown that advanced liver disease due to NAFLD will continue to place a significant burden on our healthcare system and is, therefore, an area that the medical community should continue to focus on, especially regarding preventative and therapeutic efforts.

## References

[1] Adams LA, Anstee QM, Tilg H, Targher G (2017). Non-alcoholic fatty liver disease and its relationship with cardiovascular disease and other extrahepatic diseases. Gut.

[2] Sao R, Aronow WS (2018). Association of non-alcoholic fatty liver disease with cardiovascular disease and subclinical atherosclerosis. Arch Med Sci.

[3] Keskin M, Hayıroğlu Mİ, Uzun AO, Güvenç TS, Şahin S, Kozan Ö (2017). Effect of nonalcoholic fatty liver disease on in-hospital and long-term outcomes in patients with ST-segment elevation myocardial infarction. Am J Cardiol.

[4] Xia W, Yang N, Li Y (2020). Analysis of risk factors for adverse cardiovascular events in elderly patients with acute myocardial infarction and non-alcoholic fatty liver disease (NAFLD). Med Sci Monit.

[5] (2021). Healthcare Cost and Utilization Project (HCUP). http://www.hcup-us.ahrq.gov/nisoverview.jsp.

[6] Ballestri S, Lonardo A, Bonapace S, Byrne CD, Loria P, Targher G (2014). Risk of cardiovascular, cardiac and arrhythmic complications in patients with non-alcoholic fatty liver disease. World J Gastroenterol.

[7] Alexander M, Loomis AK, Fairburn-Beech J (2018). Real-world data reveal a diagnostic gap in non-alcoholic fatty liver disease. BMC Med.

[8] Boursier J, Shreay S, Fabron C, Torreton E, Fraysse J (2020). Hospitalization costs and risk of mortality in adults with nonalcoholic steatohepatitis: analysis of a French national hospital database. EClinicalMedicine.

[9] Mantovani A, Ballestri S, Lonardo A, Targher G (2016). Cardiovascular disease and myocardial abnormalities in nonalcoholic fatty liver disease. Dig Dis Sci.

[10] Wong CR, Lim JK (2018). The association between nonalcoholic fatty liver disease and cardiovascular disease outcomes. Clin Liver Dis (Hoboken).

[11] Oni ET, Agatston AS, Blaha MJ (2013). A systematic review: burden and severity of subclinical cardiovascular disease among those with nonalcoholic fatty liver; should we care?. Atherosclerosis.

[12] Younossi Z, Anstee QM, Marietti M (2018). Global burden of NAFLD and NASH: trends, predictions, risk factors and prevention. Nat Rev Gastroenterol Hepatol.

[13] Ismaiel A, Dumitraşcu DL (2019). Cardiovascular risk in fatty liver disease: the liver-heart axis-literature review. Front Med (Lausanne).

[14] Santos RD, Nasir K, Conceição RD, Sarwar A, Carvalho JA, Blumenthal RS (2007). Hepatic steatosis is associated with a greater prevalence of coronary artery calcification in asymptomatic men. Atherosclerosis.

[15] Ma J, Hwang SJ, Pedley A (2017). Bi-directional analysis between fatty liver and cardiovascular disease risk factors. J Hepatol.

[16] Targher G, Byrne CD, Lonardo A, Zoppini G, Barbui C (2016). Non-alcoholic fatty liver disease and risk of incident cardiovascular disease: A meta-analysis. J Hepatol.

[17] Lu DY, Saybolt MD, Kiss DH, Matthai WH, Forde KA, Giri J, Wilensky RL (2020). One-year outcomes of percutaneous coronary intervention in patients with end-stage liver disease. Clin Med Insights Cardiol.

[18] Chalasani N, Younossi Z, Lavine JE (2018). The diagnosis and management of nonalcoholic fatty liver disease: practice guidance from the American Association for the Study of Liver Diseases. Hepatology.

[19] Musso G, Gambino R, Cassader M, Pagano G (2010). A meta-analysis of randomized trials for the treatment of nonalcoholic fatty liver disease. Hepatology.

